# The Invasive Region of Glioblastoma Defined by 5ALA Guided Surgery Has an Altered Cancer Stem Cell Marker Profile Compared to Central Tumour

**DOI:** 10.3390/ijms18112452

**Published:** 2017-11-18

**Authors:** Stuart J. Smith, Mohammed Diksin, Saachi Chhaya, Shwetha Sairam, Maria A. Estevez-Cebrero, Ruman Rahman

**Affiliations:** Children’s Brain Tumour Research Centre, School of Medicine, University of Nottingham, Nottingham NG7 2UH, UK; stuart.smith@nottingham.ac.uk (S.J.S.); mohammed.diksin@nottingham.ac.uk (M.D.); mzysc9@exmail.nottingham.ac.uk (S.C.); mzyss26@exmail.nottingham.ac.uk (S.S.); maria.estevezcebrero@nottingham.ac.uk (M.A.E.-C.)

**Keywords:** 5ALA, intra-tumour heterogeneity, invasion, hypoxia, glioma stem cell

## Abstract

Glioblastoma, a WHO grade IV astrocytoma, is a highly aggressive and heterogeneous tumour that infiltrates deeply into surrounding brain parenchyma, making complete surgical resection impossible. Despite chemo-radiotherapy, the residual cell population within brain parenchyma post-surgery causes inevitable recurrence. Previously, the tumour core has been the focus of research and the basis for targeted therapeutic regimes, which have failed to improve survival in clinical trials. Here, we focus on the invasive margin as defined by the region with 5-aminolevulinic acid (5ALA) (GliolanTM) fluorescence at surgery beyond the T1 enhancing region on magnetic resonance imaging (MRI). This area is hypothesized to constitute unique microenvironmental pressures, and consequently be molecularly distinct to tumour core and enhancing rim regions. We conducted hematoxylin and eosin (H&E), array real time polymerase chain reaction (PCR), and immunohistochemistry staining on various intra-tumour regions of glioblastoma to determine molecular heterogeneity between regions. We analyzed 73 tumour samples from 21 patients and compared cellular density, cell proliferation, and the degree of vascularity. There is a statistically significant difference between the core, invasive margin and other regions for cell density (*p* < 0.001), cell proliferation (*p* = 0.029), and vascularity (*p* = 0.007). Aldehyde dehydrogenase 1 (ALDH1) and Nestin immunohistochemistry were used as a measure of stem-like properties, showing significantly decreased Nestin expression (*p* < 0.0001) in the invasive margin. Array PCR of the core, rim, and invasive regions showed significantly increased fibroblast growth factor (FGF) and ALDH1 expression in the invasive zone, with elevated hypoxia inducing factor 1-alpha (HIF1α) in the rim region, adjacent to the hypoxic core. The influence of varying microenvironments in the intra-tumour regions is a major key to understanding intra-tumour heterogeneity. This study confirms the distinct molecular composition of the heterogeneous invasive margin and cautions against purported therapy strategies that target candidate glioblastoma stem-like genes that are predominantly expressed in the tumour core. Full characterization of tumour cells in the invasive margin is critical, as these cells may more closely resemble the residual cell population responsible for tumour recurrence. Their unique nature should be considered when developing targeted agents for residual glioblastoma multiforme (GBM).

## 1. Introduction

In 2014, around 10,981 people were diagnosed with a new brain, central nervous system (CNS), or intracranial tumour, making it the ninth most common cancer in the UK (Cancer Research UK data, from www.cancerresearchuk.org). Brain tumour mortality rates have increased in the last 32 years by 15% for men and 10% for women, making it an urgent target for novel therapies in the future. Astrocytomas are the most frequent type of primary brain tumour, with glioblastoma multiforme (GBM (Grade IV astrocytomas)) being the most malignant and most common type of brain tumour in adults. Even with aggressive treatment, median survival for patients is around 15 months from diagnosis [1]. Only 6% of patients diagnosed with GBM survive for five or more years after their diagnosis. Temozolomide (TMZ) induced DNA damage can be repaired by DNA repair protein O^6^-methylguanine-DNA-methyltransferase (MGMT), facilitating resistance to the drug and recurrence of the GBM [2]. Recurrence after treatment is almost inevitable and usually happens within one year of treatment and most commonly within 2 cm from the surgical resection margin [3]. The treatment of GBM involves surgical resection to reduce tumour mass; however, it is difficult to surgically determine the extent of the infiltration of the tumour cells and to define a border, making the resection subtotal in many cases. Current advances in neurosurgical techniques, such as the use of 5-aminolevulinic acid (5ALA) derived tumour cell fluorescence, improve patient safety and enhance the maximal surgical resection, leading to an increase in progression free survival [4]. 5-ALA is an intermediate metabolite in the porphyrin bio-synthesis intra-cellular pathway. Although taken up by both cancer and healthy brain cells, its metabolism results in the accumulation of fluorescent protoporphyrin IX molecule inside tumour cells only. This enables a better visualization of regions of tumour cells that have invaded into the adjacent normal brain parenchyma, therefore allowing for more radical surgery to be achieved safely and the sampling of regions of tumour more distant from the core, where invasion into normal brain is occurring. However, complete resection remains unlikely due to the extreme level of parenchyma infiltration by the tumour cells.

Many genome-wide analyses have found genetic alterations with frequent mutations in key pathways, such as receptor tyrosine kinase signaling or p53 signaling, as well as multiple less common aberrations. It has been suggested that GBM can be classified into molecular subtypes: variants with somatic mutations in isocitrate dehydrogenase 1 (IDH1), isocitrate dehydrogenase 2 (IDH2), or tumour protein 53 (TP53); transcriptional signature; copy-number variation; amplification or mutation of epidermal growth factor receptor (EGFR), and hypermethylation of promoter regions [5]. Verhaak et al. described four molecular classifications: classical, mesenchymal, proneural, and neural [6].

RNA sequencing and histological analysis, conducted by Gill et al. [7], revealed that tissue in the tumour core displayed higher cellularity, vascularity, and necrosis, whereas tissue from the invasive margin mainly consisted of neurons and other non-neoplastic cells. The intra-tumour regions were subtyped using the Verhaak classifier system; samples taken from the core showed similarities with the proneural, classical or mesenchymal subtype, whilst samples taken from the margin showed correlation with the neural subtype. Molecular and cellular characteristics of the invasive margin could be determined by the molecular subtype of the tumour core. For example, the invasive margin of a proneural GBM contained genes that were expressed in oligodendrocyte progenitor-like cells, whilst the invasive margin of mesenchymal GBM was enriched with genes that were expressed by microglia and reactive astrocytes. This information could provide value in terms of prognosis and therapy design. However, the subtypes have different susceptibilities to treatment, and so the lack of a common vulnerable therapeutic target likely contributes to treatment failure.

A possible explanation for this intra-tumour heterogeneity is thought to be the clonal evolution of the tumour cells. Studies show that GBM tumors have genetic alterations that are common amongst different regions and some alterations that are area specific. The common mutations are considered to have an evolutionary advantage when compared to other mutations, and were proposed as “driver mutations” occurring early in pathogenesis [8]. Divergent development of the cells through sub-clonal acquisition of mutations is deemed to be a major contributor to treatment failure and recurrence. Sottoriva et al. describe GBM evolution at an individual patient level with the founding clone displaying amplification of EGFR, cyclin-dependent kinase 6 (CDK6), and mesenchymal-epithelial transition factor (MET), and a loss of cyclin-dependent kinase inhibitor 2A/B (CDKN2A/B) and phosphatase and tensin homolog (PTEN), subsequently giving rise to sub-clone populations manifesting in later mutations, such as loss of NF-1 and TP53 [9]. That certain clones represent the mesenchymal subtype whilst others resemble the proneural classification, highlight the fact that clonal selection favors the whole tumour population rather than single cells by using tumour heterogeneity as a mechanism for resistance.

Cancer stem-like cells (CSCs) are self-renewing, multipotent cells that can give rise to progenitor cells, and eventually, differentiated cancer cells that have the ability to generate tumors when xenografted into immunocompromised animals [10]. CSCs may be more resistant to chemotherapy and radiotherapy than other tumour cells, allowing them to survive and cause tumour recurrence [11]. CSC resistance to chemotherapy agents can be due to their high drug efflux capacity; indeed, a high expression of drug transporters (MDR1 transporters) has been repeatedly reported [12].

It is hypothesized that CSCs may be concentrated in close proximity to neo-angiogenic vessels in the growing and invading tumour, and, which may provide a supportive microenvironment [13]. These niches may protect the CSCs from apoptotic stimuli, where endothelial cells secrete paracrine factors that promote cell survival and self-renewal [14]. Microvascular proliferation is a hallmark characteristic of GBM, which suggests that the CSCs could benefit from these highly vascularized tumour regions. CSCs also thrive in hypoxic microenvironments of the tumour through the induction of hypoxia inducible factor 2α and other molecular regulators [15]. This relationship between blood vessels and stem cells is mutualistic: CD133+ CSCs tend to produce a higher concentration of vascular endothelial growth factor (VEGF) [16]. In order to eliminate the CSCs, these vascular niches have become a potential therapeutic target. During surgery, the central zone of tumour and accompanying niches with associated stem cells will likely be resected; however, less is known about the prevalence of CSCs in the invasive margin and their potential to re-populate the tumour. The tumour microenvironment within the tumour periphery is likely to be different to the hypoxic, necrotic core, and it may be that the characteristics of CSCs and their relationship with their surroundings, in particular the vasculature, also differ.

Many previous studies have focused on samples taken from the tumour core, consisting of “pure” tumour. However, the molecular studies discussed have shown different regions of tumour and have different profiles of molecular abnormality and differing expression profiles for known tumour markers. Although evaluation of the core, pure tumour area gives information regarding the central tumour, this is the region that is most likely to be resected when surgery is visually guided under conventional light alone. This study seeks to evaluate tumour characteristics, including levels of markers of stem-like cells in the periphery of GBM, comparing the area beyond the T1 enhancing tumour (as identified by 5ALA fluorescence, where the tumour is blending with and invading the brain parenchyma) with conventional core tumour samples. It is hypothesized that tumour cells in this area will share more characteristics with the residual tumour cells left behind after surgery that cause recurrence. By understanding and targeting cells, in particular treatment resistant CSCs that are the true source of recurrence (rather than the bulk tumour core), more effective therapeutic strategies may be developed.

## 2. Results

The cohort consisted of 14 patients that were sampled during craniotomy and resection of their GBM in a multiregional fashion giving 73 tumour samples in total, with 25 samples from the core central region of the tumour, 32 samples from the viable enhancing rim, and 16 from 5-aminolevulinic acid (5ALA) fluorescence positive areas beyond tumour enhancement as per the image guidance surgical navigation system. There were eight female and six male subjects and median age was 55 years old (26–73). Overall survival in the cohort was a median of 14.9 months (3.8–36.2 months), consistent with other published series, with a 12-month survival of 61%. Three patients had a tumour with the R132H mutation in the *IDH1* gene, with 11 patients having wild type IDH1 GBM. One patient had hypermethylated MGMT promoter, with 10 hypomethylated and three of unknown status. Twelve patients completed 60Gy radiotherapy and adjuvant temozolomide after surgery, with two patients only receiving radiotherapy. IDH status was significantly associated with extended survival (*p* = 0.04 logrank test) (Table 1).

**Figure 1:**
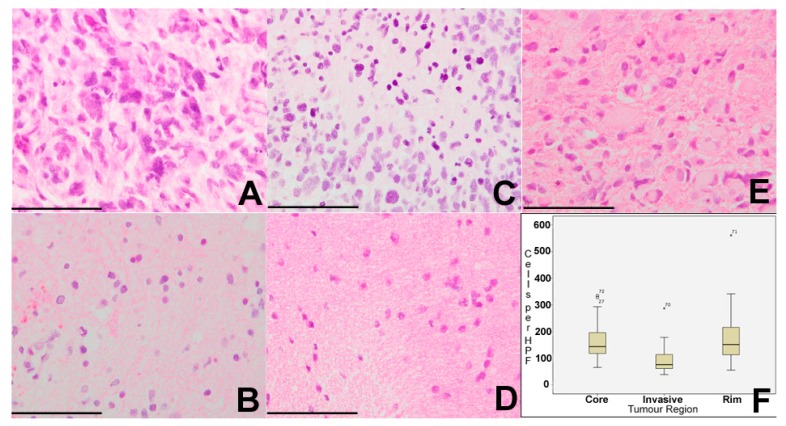
Intra-tumour cellular density reveals spatial heterogeneity. Cell density in all samples was assessed on H&E sections with a median of 127 cells per high-powered field (HPF) across all of the tumour regions (range 39–561 cells per HPF). (**A**,**B**) Tumour core. (**C**) Tumour rim. (**D**,**E**) Tumour invasive margin. (**F**) Core samples had a median cell density of 144 cells per HPF (range 66–335), rim samples a median of 152 cells per HPF (56–561) and invasive samples median of 76 cells per HPF (39–287). On pairwise post-hoc *t*-test, there were significant differences between core and invasive regions (* *p* = 0.018) and between rim and invasive regions (* *p* = 0.045) but not between core and rim regions (*p* = 0.849). (**A**–**E**) ×200. Error bars (**A**–**E**) 25 µm.

Cell density in all samples was assessed on H&E sections with a median of 127 cells per high-powered field (HPF) across all of the tumour regions (range 39–561 cells per HPF). Using the non-parametric median test, there was a significant difference in cell density between core, rim, and invasive regions of tumour (*p* = 0.031). Core samples had a median cell density of 144 cells per HPF (range 66–335), rim samples a median of 152 cells per HPF (56–561), and invasive samples median of 76 cells per HPF (39–287). On a pairwise post-hoc *t*-test, there were significant differences between core and invasive regions (*p* = 0.018), and between rim and invasive regions (*p* = 0.045), but not between core and rim regions (*p* = 0.849) (Figure 1). There was no significant correlation between cell density in any region and length of survival.

**Figure 2:**
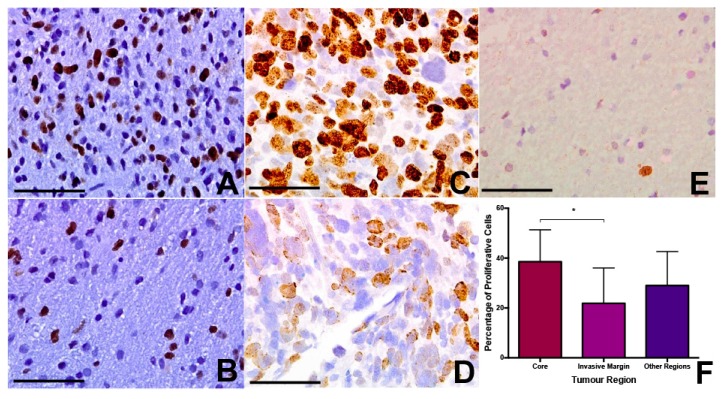
Intra-tumour proliferation index reveals lower growth rate at the GBM invasive margin. Using Ki67 immunohistochemistry we assessed the fraction of proliferating cells in each tumour region across the various samples. (**A**,**B**) Median Ki67 positive fraction was 39% for core samples (range 4–61%), (**C**,**D**) 26% for rim samples (range 2–45%) and (**E**) 22% for invasive margin samples (range 4–39%). On post-hoc *t*-test there was a significant difference between core and invasive samples (* *p* = 0.028) but not between rim and invasive or rim and core samples. (**F**) Number of cells positive on Ki67 immunohistochemistry in core, rim and invasive regions of GBM tumours. On post-hoc *t*-test, there was a significant difference between core and invasive samples, indicated by asterisk. (**A**–**E**) ×200. Error bars (**A**–**E**) 25 µm.

Using Ki67 immunohistochemistry, we assessed the fraction of proliferating cells in each tumour region across the various samples. Median Ki67 positive fraction was 39% for core samples (range 4–61%), 26% for rim samples (range 2–45%), and 22% for invasive samples (range 4–39%). This apparent trend towards a decrease in tumour proliferation in the periphery did not quite achieve statistical significance (*p* = 0.089 Kruskal-Wallis) (Figure 2). On post-hoc *t*-test, there was a significant difference between core and invasive samples (*p* = 0.028), but not between rim and invasive or rim and core samples (Figure 2). The mean level of cell density and Ki67 positivity were higher in patients not surviving six months, but this was not statistically significant (*p* = 0.132 and 0.144, respectively).

**Figure 3:**
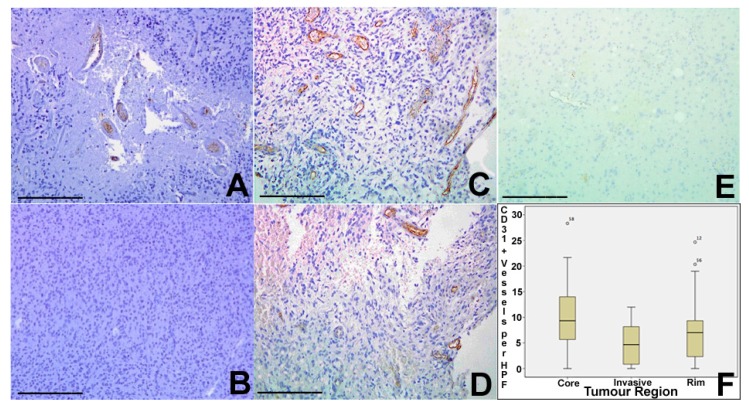
The GBM invasive margin is characterised by a distinct vasculature. Tumour vascularity was assessed using CD31 (PECAM-1) immunohistochemistry, identifying the number of vascular structures per HPF. (**A**,**B**) Core tumour regions showed a median of 9 vessels pre HPF (range 0–28), (**C**,**D**) rim regions a median of 7 vessels per HPF (range 0–25) and (**E**) invasive areas a median of 5 (range 0–12). There was statistically significant variation between areas (*p* = 0.025 Kruskal Wallis) with significant differences between core and invasive and between core and rim regions on pairwise *t*-test. (**F**) Relative levels of CD31 positivity between core, rim and invasive tumour areas. On post-hoc T-test, there was statistically significant variation between areas. (**A**–**E**) ×40. Error bars (**A**–**E**) 100 µm.

Tumour vascularity was assessed using CD31 (PECAM-1) immunohistochemistry, identifying the number of vascular structures per HPF. Core tumour regions showed a median of nine vessels pre HPF (range 0–28), rim regions a median of seven vessels per HPF (range 0–25), and invasive areas a median of 5 (range 0–12). There was statistically significant variation between areas (*p* = 0.025 Kruskal Wallis), with significant differences between core and invasive and between core and rim regions on pairwise *t*-test (Figure 3).

**Figure 4:**
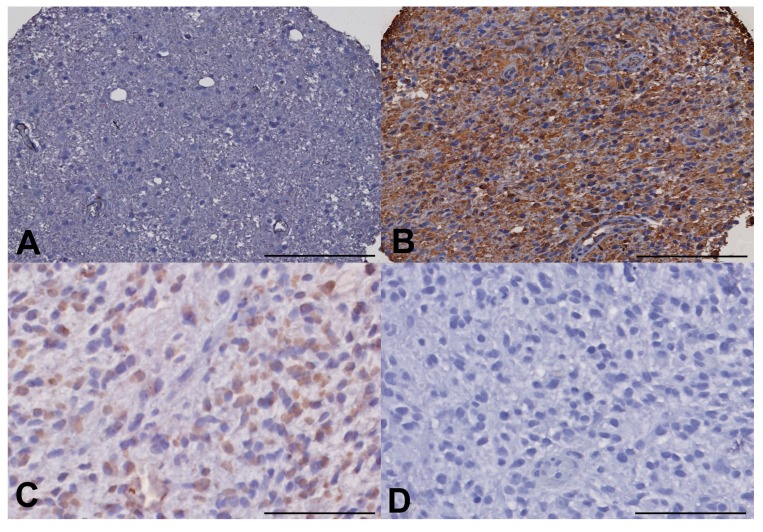
The GBM invasive margin exhibits reduced expression of canonical glioma stem cell markers. (**A**) Immunohistochemistry against nestin in invasive zone tissue showing low staining (23.1 ± 11%). (**B**) Immunohistochemistry against core tumour showing high expression of nestin (80.7 ± 31%). (**C**) Positive staining for ALDH1 in an invasive region of tumour (53.6 ± 29%). (**D**) Low levels of ALDH1 detected in the tumour core (10.3 ± 4%). (**A**,**B**) ×40, (**C**,**D**) ×200. Error bars (**A**,**B**) 100 µm; (**C**,**D**) 25 µm.

To evaluate the stemness of the GBM cell populations in different regions of the tumors, we undertook ALDH1 and Nestin immunohistochemistry, using a semi-quantified scale of increasing intensity (−,+,++,+++). ALDH1 immunohistochemistry (IHC) showed a trend toward increased expression in the fluorescent invasive region beyond the enhancing rim but this was not significant on Kruskal-Wallis (*p* = 0.202). Nestin appeared to be showing a different pattern with reduced levels of expression (none in many samples) in the invasive areas but with elevated levels in tumour core and rim areas but again this did not achieve significance (*p* = 0.212) (Figure 4).

**Figure 5:**
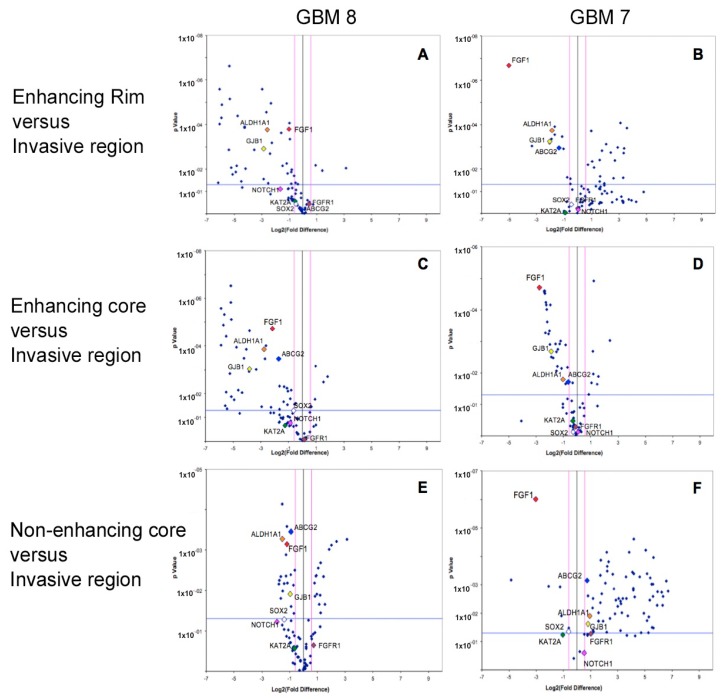
The GBM invasive margin is characterized by a unique stem cell transcriptomic profile. Array PCR studies of known stem related markers (including cell cycle regulators, chromosome and chromatin regulators, self-renewal and differentiation markers) demonstrated distinct stem cell expression profiles within different spatial regions of two primary patient GBMs (GBM 7, GBM 8). The specific stem cell markers *ALDH1A*, *ABCG2,* and *FGF1* were consistently and significantly over-expressed (all *p* values < 0.05) in the invasive margin of GBM when compared to (**A**,**B**) enhancing rim, (**C**,**D**) enhancing core and (**E**,**F**) non-enhancing core tumour regions. The gap junction protein gene *GJB1* was also consistently over-expressed in the invasive region. Other stem cell markers had varied expression between tumour regions on PCR, with expression of an average of 39 markers varying significantly between regions. Neurogenic locus notch homolog protein 1 (NOTCH1), lysine acetyltransferase 2A (KAT2A); sex-determining region Y-box 2 (SOX2); fibroblast growth factor receptor (FGFR1).

Array PCR studies of known stem related markers (including cell cycle regulators, chromosome and chromatin regulators, self-renewal and differentiation markers) demonstrated distinct stem cell expression profiles within different spatial regions of GBM, in line with the results that were observed by immunohistochemistry. The specific stem cell markers *ALDH1A* (in keeping with the IHC result), *ABCG2,* and *FGF1* were consistently and significantly over-expressed (all *p* values < 0.05) in the invasive margin of GBM when compared to rim and core tumour regions from where samples are conventionally taken. The gap junction protein gene, *GJB1,* was also consistently over-expressed in the invasive region (Figure 5). Other stem cell markers had varied expression between tumour regions on PCR, with expression of an average of 39 markers varying significantly between regions.

## 3. Discussion

Immunohistochemistry and array reverse transcriptase PCR were used in this study, aiming to assess phenotypic and genotypic cellular diversity within distinct GBM intra-tumour regions and to give an insight into the invasive margin region, which may be the most promising region for developing potential therapeutic targets to prevent the currently inevitable tumour recurrence. Our results support the hypothesis that GBMs display both inter- and intra-tumour heterogeneity specifically in terms of human stem cell gene expression, proliferative capacity, and microenvironment. In general, these results are consistent with previous work that was performed by genome-wide studies to identify tumour heterogeneity [9,17]. In particular, our profiling of 84 human stem-related genes shows a remarkable degree of GBM intra-tumour heterogeneity, which is consistent with the histological appearance of GBM, with this heterogeneity and polyclonal nature contributing to the dismal clinical prognosis. Despite this high variability in stem cell gene expression profile within different GBM regions, we have identified several consistently overexpressed genes in the invasive margin relative to other tumour regions. These genes (*FGF1*, *GJB1*, *ABCG2,* and *ALDH1A1*) could have a key role in GBM malignant infiltration through normal brain parenchyma, and may ultimately be functionally relevant to the inevitable GBM recurrence and treatment resistance despite adjuvant radio- and chemotherapy after surgical resection.

Fibroblast growth factor 1 (FGF1) ligand is secreted independently from the extracellular matrix, and it is thought to enhance the formation of branching vasculature networks [18]. Furthermore, previous work from our laboratory shows that the inhibition of VEGF and/or FGF1 pathways can block vascular mimicry [19]. Vascular mimicry in this context is the ability of cells derived from high-grade glioma tumors to generate vascular networks [20]. Vascular formation often occurs in response to reduced oxygen supply that ultimately results in the accumulation of HIF1α, which in turn stimulates the master regulator of angiogenesis VEGF, and HIF2α [21]. Alternatively, angiogenesis may sometimes be established independent to the level of oxygen via the FGF1 signaling pathway [18,22], and this could be the microenvironmental driver that we are observing in this study. It may be that upregulated *FGF1* with or without VEGF (stimulated by decreased oxygen supply through HIF1α or HIF2α) in the invasive region, could potentiate the malignant vasculature that is needed to provide the appropriate niche for malignant invasion throughout the disease course.

Furthermore, the high expression of *GJB1* that is observed at the invasive margin relative to other intra-tumour regions could also contribute to neo-angiogenesis, as this gene is known to mediate the process of endothelial cell intercommunication [23]. The regulation of *GJB1* remains complex within tumors in different tissues [24]. Our finding of elevated gene expression of *GJB1* in the invasive margin could be due to the activation of mitogen-activated protein kinase (MAPK)-dependent downstream signaling of epidermal growth factor (EGF) and FGF1 pathways. Tyrosine kinase receptor activation by EGF and FGF1 ligands has been proposed to mobilize Ca^2+^ from its intracellular stores in the endoplasmic reticulum [25]. This in turn leads to a positive feedback activation loop of *GJB1* [26]. In cancer pathogenesis, it is believed that cell fate can be regulated by intracellular Ca^2+^ waves that result in escaping apoptosis, enhancing cell migration, and promoting angiogenesis [27]. Thus, we hypothesize that the infiltrative malignant GBM cells in the invasive margin increase the intracellular Ca^2+^ gradients via GJB1 channels, which are already upregulated in response to high MAPK activity (expression of EGF and FGF1 ligands) in this region. These intracellular Ca^2+^ waves may enhance the invasive cell survival and promote their migration through normal tissue via remodeling of their actin cytoskeleton and focal adhesion [27]. Angiogenic effects of this ionic disturbance are consistent with our analysis regarding the hypoxic niche and neo-vascularization in the invasive margin of GBM. The low level of vascularity detected in the invasive area by immunohistochemistry may indicate that the neo-angiogenesis is a process in flux with the high levels of new vessels observed in the core reflecting a later effect. Although the invasion by GBM cells is well recognized to require vessel formation [28] it may be that invasion can commence with the growth of a relatively small number of new vessels initially.

Interestingly, the expression of 84 stem cell genes within different GBM regions reveals that the invasive region has the highest *ALDH1A1* and *ABCG2* expression as compared to other tumour regions. These genes have been shown to be involved in radio- and chemo-resistance, and their expression in the invasive zone could contribute to treatment resistance post-surgically [29]. Dean et al. [30] suggested that treatment resistance in tumors is due to the enrichment for stemness characteristics within certain cellular subpopulations within tumors. These cells may have a relatively slow proliferation, which allows for them to circumvent classical cytotoxic chemotherapies, which target cells with high mitotic rates. The Ki67 findings in this study indicate a highly proliferative cell population within the mass tumour, but with lower levels of mitotic activity in cells that are invading into the brain.

Stem-like cells also express ABC transporter channels, which have the ability to efflux different chemotherapeutic agents. *ALDH1A1* and *ABCG2* genes are strongly associated with the CSC phenotype [31,32]. Targeting these particular pathways after surgery could potentially decrease GBM tumour resistance.

Targeting of stem-related populations in GBM remains a significant challenge because of their cellular localization, identification, characterization, and behavioral variability. The results of our study also suggest that varying stem cell populations are located in the invasive margin and tumour mass regions, presumably in response to different micro-environmental conditions. Additionally, overexpressed *FGF1*, *GJB1*, *ABCG2*, and *ALDH1A1* stem-related genes in the invasive margin as compared to other regions of the tumors may indicate a distinct spectrum of precursor clones in the invasive margin of GBM. The stresses of prolonged hypoxia in the tumour core can stimulate the expansion of stem cell subpopulations in addition to the potentiation of reprograming non-stem glioma cells to a stem-like phenotype [33]. This plasticity of GBM, which enables them to adapt within different microenvironmental niches, suggests that combinatorial approaches will be needed to appropriately target sub-populations within different microenvironments.

In conclusion, our findings revealed considerable inter- and intra-tumour heterogeneity within distinct GBM regions. Although we observe different CSC gene expression, our invasive margin is a molecular snapshot of before the selection pressure conferred by chemo- and radiotherapy has occurred. We may not be able to exclude the possibility that CSC marker expression starts to resemble levels in the tumour core post-treatment, when self-renewal must be very active to help to repopulate the tumour. Despite the high phenotypic and genotypic cellular diversity, we have identified the potentially targeted molecular pathways in the invasive region that can be linked to GBM invasiveness and recurrence. GBM invasive behavior may be attributed to the observed *FGF1* overexpression that enhances malignant angiogenesis, which in turn provides the appropriate niche that is needed for malignant cell migration and attraction towards the normal tissue. Upregulation of *GJB1* may enhance Ca^2+^ mediated GBM cell migration by promoting endothelial intercellular communication. Similarly, *ABCG2* and *ALDH1A1* overexpression in the invasive region could be linked to stem-like subpopulations. Thus, targeting *FGF1* and *GJB1* could lead to the suppression of GBM invasion, whereas deactivation of *ABCG2* and *ALDH1A1* pathways could possibly impair GBM recurrence at the invasive margin by inhibiting the enrichment for stem-like subpopulations following chemo-radiation. Further confirmatory studies and broader molecular analysis are needed for a better understanding and efficient targeting of these molecular pathways.

## 4. Materials and Methods

### 4.1. Patient Tissue Collection

**Figure 6:**
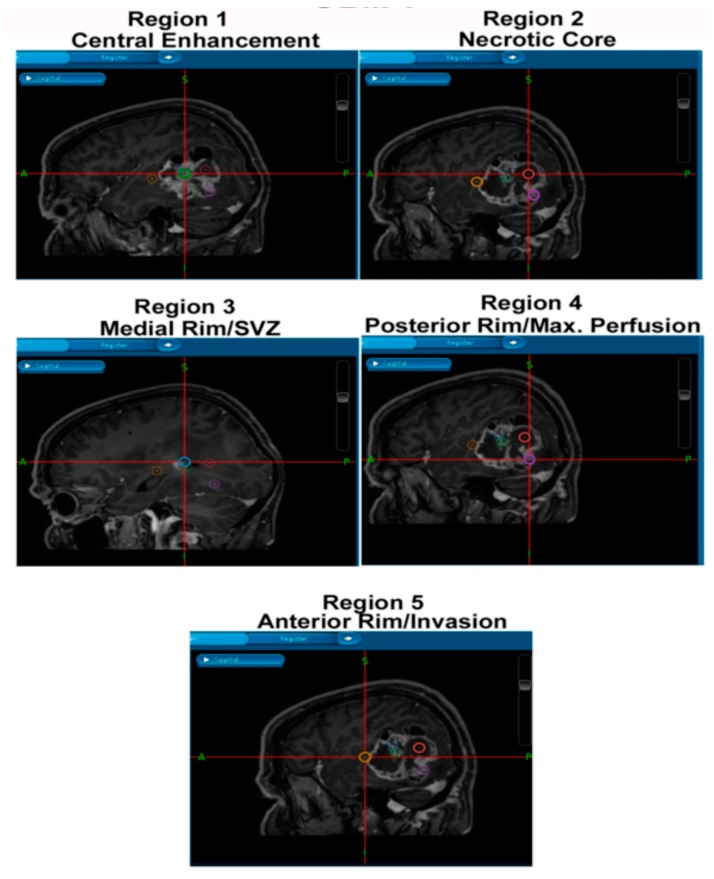
Multi-regional surgical sampling of primary glioblastoma multiforme (GBM). T1-weighted MRI scans of a representative patient, with cross-hairs depicting spatially-distinct multi-region surgical sampling. Regions include central enhancing core, necrotic core, medial and posterior rims and invasive margin (Region 5).

This project has consent and ethical approval from the Local Regional Ethics Committee in Nottingham (East Midlands Ethics Approval Reference: 11/EM/0076; 5th May 2011). Patient information is from databases within the Children’s Brain Tumour Research Centre (CBTRC), and the samples were obtained during surgeries performed by Dr Stuart Smith and colleagues in the Queen’s Medical Centre (QMC). All patients provided consent for the use of tumour samples for research and were obtained by the operating neurosurgeon. The samples were paraffin-embedded and sectioned by the Queen’s Medical Centre Histopathology Department. Samples were collected from tumour core, rim, and peripheral/invasive regions as per Figure 6.

### 4.2. Immunohistochemistry

Tonsil, pancreas, placenta, and IDH-mutant low grade glioma slides were used as controls to ensure that antibodies was specific and bound efficiently with low background staining. Tissue Microarrays (TMAs) were created within the CBTRC, and samples were separated in the TMA by tumour region and by patients.

Immunohistochemistry slides were kept at 50–60 °C overnight to fix the tissue onto the slide and to prevent tissue loss during the procedure. Next, for the removal of paraffin wax, the slide rack was placed in Xylene for 15 min, followed by absolute ethanol for 10 min, 95% ethanol for 10 min, and washed in running tap water until it clears. Samples were placed in a steamer with sodium citrate buffer (pH 6) for 40 min at 90 °C. Slides were washed in a Coplin jar with phosphate buffered solution (PBS) for 2 min, and then placed in a humidified box. 200 μL of peroxidase blocking solution was applied to cover the specimen for 5 min and then washed with PBS for 5 min. Slides were dried, then primary antibody applied diluted in antibody diluent (DAKO, Ely, Cambridgeshire, UK) (1:50 dilution for Ki-67 (DAKO) and CD31 (DAKO) and 1:300 dilution for ALDH1 (BD Transduction, San Jose, California, USA) and incubated for 1 h at room temperature. 

Sections were washed with PBS for 5 min and the secondary antibody (DAKO) was applied to cover the specimen and incubated at 37 °C for 30 min. Sections were washed again with PBS for 5 min and DAB solution made up (DAB and DAB buffer in a 1:50 dilution). The substrate-chromogen solution (DAB) was applied to cover the specimen, incubated for 5 min, and rinsed gently with distilled water. To counterstain, the slides were agitated briefly in filtered Harris hematoxylin for 10 s and washed in running water. Slides were placed in Lithium carbonate to blue for 10 s and were washed in running water before dehydrating the tissue in alcohols: 95% ethanol for 2 min and 30 s, absolute ethanol and xylene for 1 min each. Slides were mounted with DePeX medium onto a suitably sized coverslip.

The Olympus BX41 light microscope was used to view the staining and to also capture the photographs of the regions. Three representative photos were taken per slide (per region). The intensity and proportion of positively stained cells was calculated by averaging counts of three representative images of every region. All of the counting was done blindly. Areas with large amounts of necrosis and burst blood vessels were not counted, as these areas were not deemed viable and would display inaccurate, non-specific staining.

SPSS Statistics and GraphPad Prism V7.02 was used to perform two-way ANOVAs with Tukey’s multiple comparisons tests on data for each antibody and for cellular density to look for a significant statistical difference between the mean values for each region. Comparisons were made between the core and rim, rim and invasive margin, and core and invasive margin. Protein expression and cellular density was also compared between invasive and non-invasive regions.

### 4.3. Array Reverse Transcriptase PCR

RNA was extracted using *mir*VANA^TM^ miRNA Isolation Kit (Ambion; Life Technologies, Paisley, UK) according to the manufacturer’s instructions. RNA was extracted from each specimen in triplicate and all of the RNA samples were assessed for purity and quantity by using Nanodrop spectrophotometry. cDNA was synthesized by using 1μg RNA from each sample according to the manufacturer’s instructions for the cDNA synthesis RT^2^ First Strand Kit (Qiagen; Manchester, UK). Gene expression profiling was performed using Human Stem Cell PCR Array (PAHS-405Z; RT2 ProfilerPCR; Qiagen) to assess 84 human stem-related genes based on published literature and selected by the manufacturer [34]. The array is composed of 84 stem cell genes, five housekeeping genes, three positive reverse transcription controls, and one for genomic contamination control. Optimized DNA primers for real-time PCR were fixed at the bottom of each well of the 96-well array, where each primer amplifies cDNA, representing one stem cell gene. The synthesized cDNA of each sample was mixed with the PCR mastermix (RT2 SYBR^®^ Green ROXTM qPCR; Qiagen) and high-quality nuclease free water, and then loaded equally (25 μL) into each well of the stem cell array. The arrays were run in a MX3005P RT-PCR machine. The cycle conditions for array RT PCR were 95 °C for 10 min, followed by 40 cycles of 95 °C for 15 s, and 60 °C for 1 min.

After confirming the equal fluorescence detection threshold for all of the arrays in the Array-PCR machine, the obtained array data was analyzed using the accompanying Excel based statistical software that was provided by the same company (SABiosciences, Qiagen), and the significance of the fold changes was further confirmed and explored by SPSS software. Gene expression data was presented using volcano plots, in which the log fold of gene expression changes is directly plotted against the statistical significance. The fold-change threshold of gene expression was considered valid only if it was more or less than (1.5). *p*-Values were considered to be significant where less than 0.05.

## Figures and Tables

**Table 1:** Clinical data for GBM patients. All 14 GBMs presented as primary tumour cases. WT, wild-type; MT, mutant.

Tissue Sample Number & Region	Sex	Age	Tumour Site	Resection Status	Treatment RT/CT	IDH-1	MGMT Status	6 Month Survival	12 Month Survival
GBM 8	F	54	Left frontal	90%	60 Gy/temozolomide (TMZ)	WT	Not available (N/A)	No	No
GBM 9	M	48	Left frontal intrinsic	100%	60Gy/TMZ	WT	N/A	N/A	N/A
GBM 10	M	64	Right temporal	100%	60Gy/TMZ	WT	N/A	No	No
GBM 12	F	46	Left parietal	100%	N/A	WT	Hypo	N/A	N/A
GBM 13	M	26	Right parietal	100%	60Gy/TMZ	WT	Hypo	Yes	N/A
GBM 15	F	33	Right temporal	100%	60Gy/TMZ	R132H MT	Hypo	Yes	Yes
GBM 16	M	45	Left parietal	99%	N/A	R132H MT	Hypo	N/A	N/A
GBM 17	F	73	Left frontal	100%	30Gy	WT	Hypo	No	N/A
GBM 19	M	68	Left parietal	100%	N/A	WT	Hypo	N/A	N/A
GBM 20	M	71	Right frontal	99%	60Gy/TMZ	WT	Hypo	N/A	N/A
GBM 21	F	67	Right temporal	90%	60Gy/TMZ	WT	Hyper	N/A	N/A
GBM 22	F	35	Left temporal	95%	N/A	R132H MT	Hypo	N/A	N/A
GBM 25	F	60	Left parietal	99%	N/A	WT	Hypo	N/A	N/A
GBM 26	F	56	Left temporal	100%	60Gy/TMZ	WT	Hypo	Yes	No
